# New Materials and Technologies for Wastewater Treatment

**DOI:** 10.3390/ma15051927

**Published:** 2022-03-04

**Authors:** Lionel Limousy, Thomas Thiebault, Jocelyne Brendle

**Affiliations:** 1 Institut de Science des Matériaux de Mulhouse, UMR CNRS 7361, Université de Haute-Alsace, Université de Strasbourg, 3b Rue Alfred Werner, F-68100 Mulhouse, France; jocelyne.brendle@uha.fr; 2 Ecole Pratique des Hautes Etudes, UMR CNRS METIS 7619, PSL Université, 4 Place Jussieu, F-75252 Paris, France; thomas.thiebault@ephe.psl.eu

## 1. Introduction

This Special Issue (SI) contains the successful submissions [1,2,3,4,5,6,7,8,9,10,11] of Materials on the subject area of “New Materials and Technologies for Wastewater Treatment”. The development of new materials that are able to enhance the efficiency of industrial wastewater treatment processes, as well as the modulation of these materials’ properties to improve the recovery of pollutants, has experienced steady progress. Anthropogenic activities such as mining, industry, and electricity production generate secondary effluents containing various contaminants, such as heavy metals, radionuclides, dyes, etc. In view of their potential impact on water quality, the design of novel technologies aiming at recovering those contaminants is of high concern. Among the various techniques used in water pollution control, adsorption is considered to be a relevant technique because of its simple design, universal nature, high effectiveness, and ease of operation and regeneration. The theme of the SI has been devoted to the development of new materials (bulk, composites, and hybrids) through the improvement/transformation of specific processes for wastewater treatment. The improvement of AOPs (Advanced Oxidation Processes) processes has also been highlighted.

We have accepted papers presenting research findings in the development of innovative materials for the removal of sludges and of soluble forms of surfactant, dyes, phosphate and radionuclides. Beyond the scientific novelty of the suggested materials, the authors have emphasized the potential of their materials/technologies to implement full-scale facilities operating under realistic conditions. Both laboratory and pilot-scale experimental works have been considered. This Special Issue has evaluated papers coming from different fields of research: material science, chemical engineering and processing, chemistry, and biochemistry in particular.

This Special Issue contains eight research papers and three reviews. Authors’ geographical distribution (published papers) is:

- Poland (4)
- China (1)
- Canada (1)
- France (1)
- Romania (1)
- Slovakia (1)
- Spain (1)
- Egypt (1)

Published submissions are related to new adsorbents (ceramsite, multi-walled carbon nanotubes, LDHs, biopolymers, ion imprinted polymer, etc.), new materials (iron carbon substrate, nanoparticles doped polymer, phyllosilicate containing iron) and processes (AOPs). The different papers show the diversity of research conducted on wastewater treatment.

## 2. Short Review of the Contributions in This Issue

Materials in water treatment are mainly used as adsorbents for a wide variety of targets, but not only for this use. For example, Czarnota et al. assessed the potential of powdered ceramsite and limestone to improve the settling of aerobic granular sludges, and their impact on the removal of classical wastewater parameters [1]. By monitoring the granule formation and shape for a total time of three months, the authors exhibited that despite the fact that the granulation process was not complete, both powdered ceramsite and limestone acted as a ballast for the formation of sludge flocs. Moreover, the presence of powdered ceramsite at 3.0 g L^−1^, and to a lesser extent, powdered limestone, improved the removal efficiencies of chemical oxygen demand (COD), total nitrogen and phosphorus in the synthetic wastewater used.

The other contributions to this Special Issue used materials in a more conventional way, i.e., as adsorbents. The adsorbent’ types are distributed from raw geosorbents to synthetic adsorbents, meeting the specific needs of certain types of industrial water treatment, for surface water treatment, or for domestic water treatment in general.

Current water treatment technologies are considered inadequate for a complete removal of some inorganic contaminants such as trace metals, especially industrial wastewater. Layered double hydroxides (LDHs) represents a promising adsorption solution, especially for cationic ions as Mn(II). Mg-Al and Mg-Al-Ni LDHs were synthetized by the co-precipitation method in the study of Modrogan et al., with the aim of removing Mn^2+^ from synthetic wastewater [2]. Both LDHs exhibited non-porous and well crystallized structures as testified by XRD (X-ray Diffraction) and SEM (Scanning Electron Microscopy) analyses. The authors further optimized some parameters (i.e., temperature, contact time, starting concentration) and the most interesting results were that increasing temperature (i.e., 283.15 and 193.15 K tested) weakly increased the adsorption capacity, although the adsorption capacity of Mg-Al LDH was twice that of Mg-Al-Ni LDH. Kinetic experiments revealed an equilibrium time close to 60 min for both LDHs, which is a crucial parameter to consider when implementing such technology in a dynamic context.

Nutrients, and especially phosphorus, are important elements for biological processes and are extensively used in this aim as fertilizers. However, excessive concentrations in environmental compartments such as surface waters lead to eutrophication. Therefore, it is important to both develop a rational use of such fertilizers and optimized wastewater treatment technologies. Phosphorus is mostly removed through adsorption/precipitation during primary settling, improved by the presence of coagulants in order to accelerate the settlement. Yet, in order to optimize this adsorption/precipitation process for the phosphorus removal, alternative adsorbents may be considered. This is the main objective of the manuscript by Gubernat et al., in which the adsorption properties (mostly adsorption capacity) of 25 types of adsorbents were reviewed, from raw geosorbent as bentonites, to waste adsorbent [3]. The main findings of their study were that for natural adsorbents, the higher adsorption capacities were observed for carbonate derived sorbents (i.e., marble, opoka) and that a calcination strongly increased the adsorption capacity through calcium carbonate decomposition. This calcination step is, however, energy consuming and raises alkalinity issues. Finally, commercial products were assessed, and two existing materials, corresponding to calcinated opoka, were presented as the most capacitive adsorbents which can be promising for both the adsorption of phosphorus from wastewater and the further release for fertilization purpose.

Maximizing the solid/liquid ratio is often a good way in order to enhance the adsorption extent. In water treatment, constructed wetlands are gravel packs in which several removal processes are favored due to the presence of vegetation and various types of filter beds. Zhao et al. proposed a new substrate, prepared from zeolite particles, iron powder, activated carbon and a binder (i.e., water-based polyurethane), for improving the removal of phosphorus from wastewater [4]. The authors have optimized several parameters as the most favorable carbon source, the good thickener quantity and the ideal Fe/C ratio, etc. As an interesting conclusion, the developed adsorbent exhibited the best adsorption capacity among the existing literature, but these optimized conditions necessitate acidic conditions which are not common in wastewater. However, the developed displayed consistent adsorption properties in more classical wastewater conditions.

Carbonaceous adsorbents are often described as very promising for the adsorption of various solute. The most classical type of used carbonaceous adsorbent for water treatment are activated carbons, which can be under granular or powdered forms. Yet, other types of preparation can be conducted, as illustrated in the study by Aljohani et al., in which the authors synthetized multi-walled carbon nanotubes (MWCNTs) adorned with Zn and Ag NPs (nanoparticles) [5]. The preparation of the adsorbent, through the double arc discharge method, allowed cheap production costs, a major limitation for the technology transfer from research to companies. The adsorption of MB (Methylene Blue), a dye, onto the adsorbent was further evaluated following different variables (i.e., time, pH, adsorbent dose) and classical adsorption models were applied. Interestingly, the authors also performed a reusability test, by desorbing the adsorbed MB with ethanol, and demonstrated that after five cycles of adsorption/desorption, the adsorption percentage remains very significant (i.e., >90%), and that the desorption of NPs was limited, hindering the potential toxicity of the adsorbent.

Beyond MWCNTs, NPs can be added to other type of adsorbents in order to improve their adsorption properties. Antico et al. doped polymer-inclusion membranes (PIMs) based on CTA with various amounts of SiO_2_, TiO_2_, Fe_3_O_4_ NPs, as well as MCWNTs, through a simple preparation procedure (i.e., mixing and magnetic stirring) [6]. The stability assessment displayed insignificant mass loss for NP-doped PIMs in comparison with non-doped PIMs, allowing their further use for the adsorption of phosphate and arsenate from simulated natural water. The main finding of this work is the high removal efficiency of both arsenate and phosphate for Fe_3_O_4_-doped PIMs with limited transport efficiency when receiving NaCl solution in comparison with other adsorbents, highlighting the potential of these materials for an optimized extraction of these solutes from natural waters.

The modification of polymer-based materials can be performed with NPs, as described above, but also with other compounds, such as small organic molecules, or ions. Solgi et al. described such modification of pelletized chitosan-based adsorbent, with glutaraldehyde and/or calcium for the adsorption of sulfate from saline waters in column experiments [7]. The different adsorbents were characterized before performing breakthrough curves of sulfate in 0.3 H × 0.016 i.d. columns. Beyond the selected adsorbents, bed height, flow rate and starting sulfate concentration were optimized. The results highlighted the higher adsorption performance of modified chitosan adsorbents with calcium in terms of adsorption capacity, with higher removal efficiency at pH 4.5 due to the protonation of chitosan, favoring the ion-exchange mechanism for the removal of sulfates. Finally, the reusability of this material was tested, and a decrease of the performance was observed after five cycles.

Among the polymer-based adsorbents, ion-imprinted polymers (IIPs) displayed several advantages and particularly a high selectivity. This selectivity is therefore particularly interesting for targeting the removal of hazardous ions, such as radionuclides. Such adsorbents are prepared from four basic elements, a functional monomer, a crosslinker, an initiator and an inert template. The rapid development of the research conducted on these materials resulted in a high chemical variability of each component. Therefore, Kusumkar et al. proposed a state of the art of the research on IIPs synthesis and characterization, and their use for the removal of radionuclides [8]. This review allowed the identification of some sparsely studied radionuclides (i.e., TcO_4_^-^ and Ce^3+^) that deserved a research effort, and constituted a wealth of information for researchers interested in the IIPs preparation or the selective removal of radionuclides.

The concept of flocks fragmentation and the averaging method for the application of electrocoagulation in the process of coke oven wastewater treatment has been studied by Mierzwinski et al. [9]. Electrocoagulation can be an alternative to the conventional coagulation process (chemical). The investment for such a coagulation system, even at the laboratory scale, is quite important and requires specific devices and setup which limits the possibility to obtain funding to achieve this kind of study. Then, the main drawback remains the potential cost of the system in comparison with other technics. In order to overcome this problem, mathematical and numerical simulations allow for cheaper approaches to evaluate the potential gain of such complex and expensive processes. The main information of this study concerns the real potential of the complex system that has been designed and patented, which enables improvement of the treatment of coke oven wastewater. Of course, these results need to be confirmed experimentally.

Advanced oxidation processes (AOPs) have been widely studied and developed during the past ten years. The paper of Cipriano Crapiano et al. [10] is an outstanding review on clay materials used as electrodes in the electro-Fenton process. The core of this review concerns the catalytic activity of iron within the lattice of clay minerals. First, the localization of iron in the clay structure is discussed and the impact on the phyllosilicate group is shown. The catalytic activity of structural iron II towards H_2_O_2_ and O_2_ is described and the combination of these materials to electrochemical systems gives a good overview of the potential of iron modified clays to be used in the AOP process.

The last paper of this Special Issue relates to new developments for laundry wastewater treatment, and particularly the coupling of a moving bed bioreactor with photolysis oxidation and membrane cascade (MF + NF) [11]. The in-situ generation of O_3_ using UV can be an attractive and performing system to improve the degradability of anionic and non-ionic surfactants. The main interest concerns the reusability of water in such systems, which imposes a very high quality and the absence of organic residue. The work carried out by Mozia et al. gives some interesting information about the feasibility and the interest of coupling UV/O_3_ oxidation to a moving bed biological reactor (MBBR), followed by microfiltration and nanofiltration. Besides the decrease of BOD_5_ and COD in the wastewater stream, the permeation flux of the microfiltration process is improved. The only point that needs to be solved concerns the concentration of inorganic contaminants that should be treated in order to reach a high-water quality.
